# Therapeutic Effects of Human Mesenchymal Stem Cells in a Mouse Model of Cerebellar Ataxia with Neuroinflammation

**DOI:** 10.3390/jcm9113654

**Published:** 2020-11-13

**Authors:** Youngpyo Nam, Dongyeong Yoon, Jungwan Hong, Min Sung Kim, Tae Yong Lee, Kyung Suk Kim, Ho-Won Lee, Kyoungho Suk, Sang Ryong Kim

**Affiliations:** 1 Brain Science and Engineering Institute, Kyungpook National University, Daegu 41566, Korea; blackpyo2@naver.com (Y.N.); jungwan33@naver.com (J.H.); 2 BK21 Plus KNU Creative BioResearch Group, School of Life Sciences, Kyungpook National University, Daegu 41566, Korea; ydy10@naver.com; 3 Bioengineering Institute, Corestem Inc., Seoul 13486, Korea; mskim@corestem.com (M.S.K.); tylee@corestem.com (T.Y.L.); kskim@corestem.com (K.S.K.); 4 Department of Neurology, School of Medicine, Kyungpook National University, Daegu 41944, Korea; 5 BK21 Plus KNU Biomedical Convergence Program, Department of Pharmacology, School of Medicine, Kyungpook National University, Daegu 41944, Korea

**Keywords:** cerebellar ataxia, neuroinflammation, microglia, mesenchymal stem cell, lipopolysaccharide

## Abstract

Cerebellar ataxias (CAs) are neurological diseases characterized by loss of muscle coordination that is a result of damage and inflammation to the cerebellum. Despite considerable efforts in basic and clinical research, most CAs are currently incurable. In this study, we evaluated the therapeutic potential of human mesenchymal stem cells (hMSCs) against CAs associated with neuroinflammation. We observed that hMSC treatment significantly inhibited the symptoms of ataxia in lipopolysaccharide (LPS)-induced inflammatory CA (ICA) mice, which were recently reported as a potential animal model of ICA, through the anti-inflammatory effect of hMSC-derived TNFα-stimulated gene-6 (TSG-6), the protection of Purkinje cells by inhibition of apoptosis, and the modulatory effect for microglial M2 polarization. Thus, our results suggest that hMSC treatment may be an effective therapeutic approach for preventing or improving ataxia symptoms.

## 1. Introduction

Cerebellar ataxias (CAs) are heterogeneous neurological disorders caused by cerebellar damage that can affect balance and coordination of movement [1,2]. Some studies have reported that the application of targeted therapies to cerebellar ataxia with vitamin E deficiency (AVED) and gluten ataxia suppresses disease progression and improves neurological function. However, most CAs cannot be cured and symptom alleviation is the only option [3,4,5]. CAs are diverse and complex in their causes, including acquired conditions (for example, toxic agents, immune-mediated inflammation, infection, chronic alcohol, and vitamin deficiencies), sporadic neurodegenerative disorders, genetic defects, and unknown reasons [1,3,6]. In addition, many types of CA have been reported to be closely related to cerebellar inflammation [1,3,7]. Recent studies demonstrated over-activation of glial cells and production of proinflammatory molecules in the cerebellum of patients with CA and animal models of CA, caused by genetic mutations, viral infections, and toxic exposures [8,9,10,11,12]. Moreover, several studies have indicated that cerebellar inflammation can lead to the loss of Purkinje cells, resulting in cerebellar dysfunction [1,13]. However, there is currently no appropriate animal model to study CA associated with cerebellar inflammation. Therefore, we have recently developed an inflammatory CA (ICA) animal model induced by lipopolysaccharide (LPS) injected directly into the cerebellum [14].

Human mesenchymal stem cells (hMSCs) are multipotent cells, which have the ability to self-renew and differentiate into multiple specialized cells [15]. In the damaged brain, hMSCs selectively target injured regions and secrete a variety of growth factors, cytokines, and chemokines that are known to have a wide range of activities, such as anti-apoptosis, angiogenesis, anti-inflammation, immunomodulation, and chemoattraction [16,17,18,19]. hMSCs may have therapeutic potential against neurological disorders, such as Alzheimer’s disease, Parkinson’s disease, and stroke [20,21,22]. Some reports suggest that treating CA with hMSCs could attenuate the motor dysfunction caused by gene mutations and drug exposure [23,24,25,26]. However, there is little information about the effects of hMSCs in neuroinflammation-related CA. In the present study, to explore the effects of hMSCs in CAs associated with cerebellar inflammation, we examined whether treatment with hMSCs could induce therapeutic effects against LPS-induced neurotoxic events in ICA mice.

## 2. Materials and Methods

### 2.1. Experimental Design

The aim of this study was to examine the therapeutic potential of hMSCs for LPS-induced ICA model mice [14]. Animal experiments were conducted to investigate the beneficial effects of hMSCs in the LPS-induced ICA mice by using three to five mice per each group. These mice were housed in a controlled environment (12 h light/12 h dark condition) with ad libitum access of water and food. All experimental animal procedures were performed in accordance with approved animal protocols and the guidelines of the Animal Care Committee of Kyungpook National University (No. KNU 2019–0002). All efforts were made to minimize both the suffering and the number of animals involved in the experiments.

### 2.2. ICA Animal Model and hMSCs Transplantation

Inflammatory CA was induced by injection of 5 μg LPS in 5 μL of phosphate-buffered saline (PBS) directly into the cerebellum, as described in a previously published procedure with minor modifications [14,27]. Male C57BL/6 mice (approximatedly 20–25 g, 10 weeks old, Daehan Biolink Co., Ltd., Eumseong, Korea) were anesthetized via intraperitoneal administration of a mixture of ketamine (115 mg/kg, Yuhan Corporation, Seoul, Korea) and Rompun (23 mg/kg, Bayer Korea Ltd., Seoul, Korea). After placing the mouse on a stereotaxic frame (David Kopf Instruments, Tujunga, CA, USA), mice were injected with 5 μL of LPS (1 mg/mL) or phosphate buffered saline (PBS) into the cerebellum (AP: −2.5 mm; ML: 0.0 mm; DV: −2.5 mm, relative to the lambda) through a syringe pump (KD Scientific, New Hope, PA, USA) connected to the Hamilton syringe (10 μL, 30-gauge). To minimize the reflux along the injection tract, the syringe was left in place for additional 5 min.

For hMSCs transplantation, LPS-injected mice were randomly grouped before those transplantation. The head of the mouse was repositioned at an angle of approximately 90° to the body in a stereotaxic frame. After exposure of the underlying dura mater, hMSCs (1 × 10^5^ cells/20 μL or 1 × 10^6^ cells/20 μL) were transplanted into the cisterna magna using a Hamilton syringe (25 μL, 30-gauge) attached to a syringe pump (2 μL/min). The needle was removed 10 min later, and the incision was closed with silk suture. For the evaluation of tumor necrosis factor alpha-stimulated gene-6 (TSG-6)-induced effects in the LPS-treated ICA mice, recombinant human TSG-6 (rhTSG-6; 0.1, 0.5 and 2.5 μg/20 μL; R&D systems, Minneapolis, MN, USA) was introduced with the same method into the cisterna magna using a Hamilton syringe.

### 2.3. hMSC Isolation and Characterization

Human bone marrow (BM) samples were obtained from eight healthy donors according to the reviewed and approved protocols of the Institutional Review Board of Chilgok Kyungpook National University Hospital (IRB: 2017-11-015-007).

Mononuclear cells were isolated from the BM by density gradient centrifugation using Ficoll (Ficoll-Paque Premium; GE Healthcare Bio-Sciences AB) [28], seeded at a density of 11.5 × 10^7^ cells/cm^2^ and cultured in CSBM-A06 medium (Corestem, Inc., Seoul, Korea) with 10% fetal bovine serum (Life Technologies, Grand Island, NY, USA), 2.5 mM L-alanyl-L-glutamine (Biochrom AG, Berlin, Germany), and 1% penicillin-streptomycin (Biochrom AG). Non-adherent cells were removed by washing with fresh medium, and media were changed twice a week. The adherent cells grown to 70–80% confluence were defined as passage zero (P0). The cells were subcultured to passage 10 (P10) before experiments.

Population doubling time (PDT) was measured in a continual subculture and calculated using the formula: (T − T0)∙log2/log(N − N0), where T0 is the cell seeding time, T is the time at cell harvest, N0 is the initial number of cells, and N is the number of harvested cells [29]. Differentiation was induced according to previously described protocols [30]. In brief, hMSCs were cultured into 24-well plates and stimulated to differentiate along adipogenic, osteogenic, and chondrogenic lineages using an hMSC functional identification kit (R&D Systems, Minneapolis, MN, USA). After differentiation, lipid droplets in adipocytes were visualized with oil red O staining (Sigma, Saint Louis, MO, USA). Osteogenic differentiation was assessed based on the staining of calcium deposits by alizarin red (Sigma, Saint Louis, MO, USA), whereas chondrogenic differentiation was determined by alcian blue staining (Sigma, Saint Louis, MO, USA). To confirm the hMSC characteristics, cells within five passages were stained with the cell surface markers CD29, CD73, CD90, CD105, CD34, CD45 (BD Pharmingen, Heidelberg, Germany), and CD44 (BD Biosciences, San Diego, CA, USA) [31]. The expression of cell surface markers was measured using a flow cytometer (BD FACS Canto™ II), and hMSCs were identified as CD29/CD44/CD73/CD105-positive and CD34/CD45-negative cells.

### 2.4. Behavioral Tests

Behavioral tests were performed on day 1, before hMSC transplantation. Rotarod tests were performed weekly for 4 weeks and behavior was evaluated using the simple composite phenotype scoring system for a single time point at 4 weeks post-hMSC transplantation.

Rotarod tests were conducted to examine the locomotor coordination and balance of mice, as has been previously described [25]. All mice were trained for behavior analysis experiments as follows. Each trial lasted a total of 10 min. Each experimental mouse was placed on the rotarod apparatus (3 cm rod diameter), which was accelerated from 4–40 rpm over the course of 5 min and remained at 40 rpm for an additional 5 min. Training was repeated 3 times to acclimate the mice. After the last training session, mice who did not complete a total of 10 min on the rotarod were excluded from the experiment and mice who completed training were randomly divided into groups for experimental trials. Behavioral analysis experiments were conducted three times per week, with a 10 min rest period included between trials to avoid stress and muscle fatigue. The latency of time elapsed before each mouse fell from the accelerating rod was recorded. The results of these behavioral experiments were expressed as the time prior to fall from the rotarod averaged over three experimental replicates.

The simple composite phenotype scoring system, consisting of ledge test and hindlimb clasping test, was utilized to quantify disease severity of the LPS-injected mouse model of CA. Tests were performed as previously described with some modification. The scoring system was quantified at 14 weeks of age. Both the ledge test and hindlimb clasping test were scored from 0 to 3, with 0 indicating an absence of relevant symptoms and 3 indicating extremely severe symptoms, with each score summed to yield a combination phenotype of 0 to 6. To ensure the reproducibility of each score, each test was performed in triplicate.

The ledge test was used to score the degree of imbalance and lack of coordination of the mouse model of ICA by evaluating the ability of mice to walk along the ledge of a cage. Mice that walked normally along the ledge of a cage without losing their balance received a score of 0. Mice walking along the cage ledge with asymmetrical posture received a score 1. A loss of footing while walking along the ledge of a cage caused a mouse to receive a score 2. Finally, when a mouse did not effectively use its hind legs while walking, it received a score of 3.

Hindlimb clasping test was used as a marker of disease progression in mouse models of CA and consisted of scoring the mouse according to the hindlimb position of mice when they were lifted up. A score of 0 corresponded to sustained spreading of hindlimbs away from the abdomen. If one of the hindlimbs was retracted towards the abdomen for more than half of the test length, the mouse received a score of 1. Mice with both hindlimbs partially retracted towards the abdomen for more than half of the test received a score of 2. Finally, when both hindlimbs were completely retracted and touching the abdomen for more than half of the test duration, the mouse received a score of 3. Quantitative analyses of all behavioral responses were performed blind.

### 2.5. Carboxyfluorescein Succinimidyl Ester (CFSE) Labeling of hMSCs for Transplantation and Biodistribution of hMSCs in the Inflamed Cerebellum

Before transplantation, hMSCs were labeled with the tracing dye CFSE (Abcam, Cambridge, UK), as described previously [32], to investigate the ability of hMSCs to migrate toward the inflamed cerebellum. Briefly, after 15 min of incubation with 20 mM CFSE at 37 °C, the cells were washed three times with PBS following with incubation in DMEM for 30 min to allow the unbound CFSE diffuse out of the cells to prevent leakage of subsequent dye from the transplanted fluorescent hMSCs to adjacent cells. Subsequently, LPS-injected mice received 1 × 10^6^ cells in 20 μL of HypoThermosol (HTS) via the cisterna magna. One day or seven days post-transplantation, brains were harvested and sectioned coronally at a thickness of 30 μm. To investigate the biodistribution of hMSCs, the serial sections from the cerebellum that contained CFSE-labeled hMSCs were stained with 4′,6-diamidino-2-phenylindole (DAPI) and scanned using a slide scanner (Pannoramic SCAN; 3DHISTECH Kft, Budapest, Hungary). The CFSE-positive areas were measured using Multi Gauge V3.0 software (Fujifilm Life Science, Tokyo, Japan). Quantitative data were obtained from 4 sections per mouse and 4 mice per group. Further images were acquired with a confocal microscopy (TI-RCP; Nikon, Japan).

### 2.6. Immunofluorescence Staining

Mice were transcardially perfused with 4% paraformaldehyde (PFA; Sigma, St. Louis, MO, USA) in 0.1 M PBS under anesthesia of ketamine/xylazine mixture, as described previously [33]. The brains were removed and subsequently post-fixed in 4% PFA overnight before transferring into 30% sucrose solution for 48 h at 4 °C. By using HM525 NX cryostat microtome (Thermo Fisher Scientific, Scoresby, VIC, Australia), brains were cut into free-floating sagittal cerebellar sections (30-μm-thick) for immunofluorescence staining. Brain sections of cerebellum were washed with 0.1 M PBS, and blocked with 0.5% BSA in 0.1 M PBS for 1 h. Subsequently, sections were incubated at 4 °C for two overnight with the following primary antibodies, list of the antibodies used is provided in Table 1. After two days, sections were incubated with secondary fluorescents-labeled IgG antibody and counterstained with DAPI (Sigma) at room temperature. Slides were mounted with Vectashield mounting medium (Vector Laboratories, Burlingame, CA, USA). Each slide was examined using a fluorescence microscope (Axio Imager; Carl Zeiss, Gottingen, Germany).

### 2.7. Western Blot Analysis

Total cell lysates from the vermis of the mouse cerebellum were prepared for Western blotting, as previously described [34]. Cerebellar vermis were separated, and each tissue was homogenized in lysis buffer (58 mM Tris-HCl, pH 6.8; 10% glycerol; and 2% SDS) with a protease inhibitor cocktail (1:100; Millipore, Burlington, MA, USA) and phosphatase inhibitor cocktail (1:100, Cell Signaling Technology, Beverly, MA, USA). After centrifugation of lysates, protein concentration of the lysates was determined by using BCA kit (Bio-Rad Laboratories, Hercules, CA, USA). Proteins were separated using gel electrophoresis and transferred to membranes using an electrophoretic transfer system (Bio-Rad Laboratories, Hercules, CA, USA). Membranes were cut into the appropriate molecular weight size of each protein, and membranes were subsequently incubated overnight at 4 °C with the primary antibodies listed in Table 1. After incubation with horseradish peroxidase (HRP)-conjugated secondary antibodies (Amersham Biosciences, Piscataway, NJ, USA), blots were developed via enhanced chemiluminescence (ECL) Western blotting detection reagents (Amersham Biosciences, Piscataway, NJ, USA). Band densities were measured with a ImageQuant LAS 500 imager (GE Healthcare Life Science, Little Chalfort, UK) for semi-quantitative analyses. The intensity of each protein band was calculated by Multi Gauge V3.0 software (Fuji Film, Tokyo, Japan) and normalized to that of the corresponding β-actin band.

### 2.8. Statistical Analysis

All statistical analyses are depicted as mean ± standard deviation (SD) by using version 12.0 SigmaPlot software (Systat Software, San Leandro, CA, USA). To assess normal distribution and equal variance of data, the Shapiro–Wilk test and Brown–Forsythe test were applied. For multiple comparisons, the statistical significance of sample differences was analyzed using one-way analysis of variance (ANOVA) with Tukey’s post hoc test. In cases in which two groups were directly compared, Mann–Whitney U statistics and two-sided paired t-tests were employed.

## 3. Results

### 3.1. Characterization of hMSCs In Vitro

**Figure 1:**
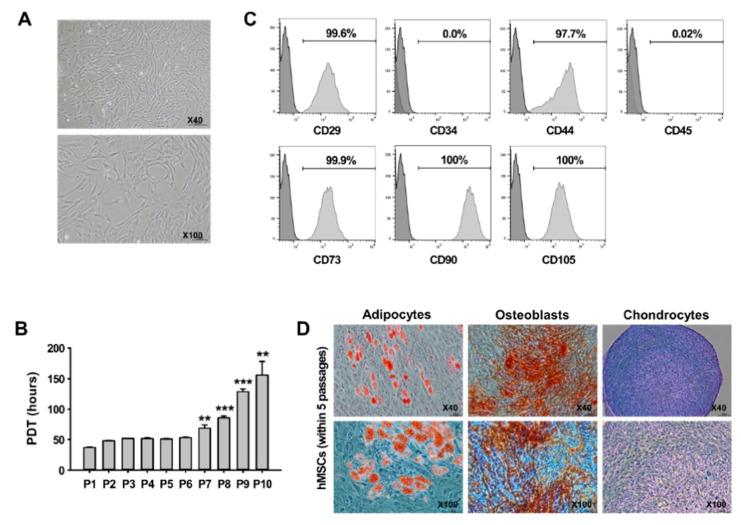
Characterization of human mesenchymal stem cells (hMSCs) derived from bone marrow (BM). (**A**) Morphology of cultured hMSCs in the early (within 5 passages, P5) phases. All hMSCs exhibited spindle-shaped morphology. (**B**) Analysis of population doubling time (PDT) of hMSCs within 10 passages. ** *p* < 0.01, *** *p* < 0.001 vs. P5 (two-sided paired t-test; *n* = 4 per group). (**C**) Immunophenotypic analysis for expression profiles of cell surface markers of hMSCs in the early phases. (**D**) Multi-lineage differentiation capacity of hMSCs. Differentiation of hMSCs into adipogenic, osteogenic, and chondrogenic lineages was stimulated then the differentiated cells stained with either oil red O (left, adipocytes), alizarin red (middle, osteoblasts), or alcian blue (right, chondrocytes).

hMSCs were characterized via fibroblastoid morphology, expression patterns of hMSC-associated surface markers, and differentiation potential, in accordance with the criteria of the International Society for Cellular Therapy (ISCT) [35]. hMSCs isolated from BM had the morphology of spindle-shaped fibroblasts in the early (within five passages, P5) and late (P6–P10) phases (Figure 1A). To evaluate the PDT of hMSCs, proliferation ability was measured according to the passage number. hMSCs in the early phases demonstrated high proliferative ability (37 h–51 h) and no significant changes between passages. However, the PDT of hMSCs in the late phases was prolonged as the number of increased passages (Figure 1B). Flow cytometry analysis revealed that more than 95% of hMSCs in the early phases were positive for the hMSC-related CD markers, such as CD29, CD44, CD73, CD90, and CD105, and were negative for the hematopoietic stem cell-related CD markers CD34 and CD45 (Figure 1C). Cells in the early phases were cultured in adipogenic, osteogenic, and chondrogenic media to investigate the differentiation capacity of the BM-derived hMSCs. Under the three specific conditions, cells were able to differentiate into multiple cell types (adipocytes, osteoblasts, and chondrocytes) (Figure 1D). Based on these results, only hMSCs within five passages were used in all experiments.

### 3.2. Migration of hMSCs into the LPS-Exposed Cerebellum after Intrathecal Implantation via the Cisterna Magna

**Figure 2:**
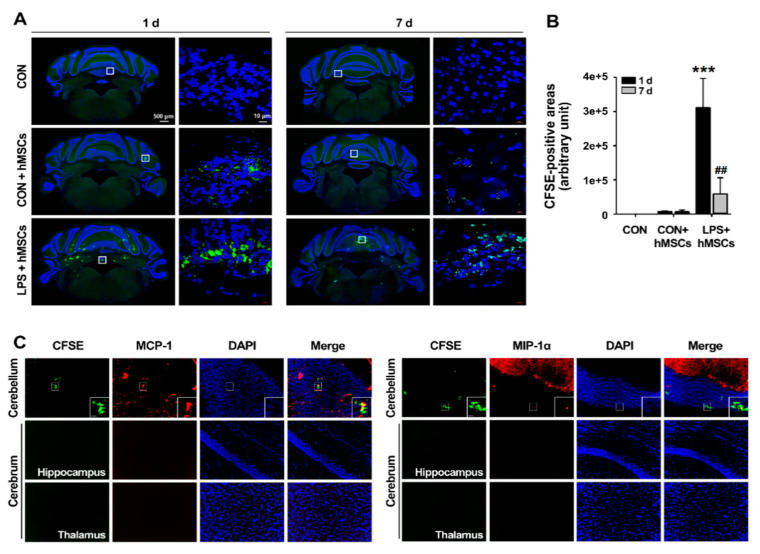
The distribution of transplanted hMSCs into LPS-exposed cerebellum after intrathecal injection. (**A**) Comparison of the overall distribution of CFSE-labeled hMSCs in the cerebellum between control and LPS-injected mice 1 day and 7 days after hMSC transplantation. Frozen cerebellum section shows double staining with the DAPI nuclear stain and CFSE (green) (Scale bar = 500 or 10 μm). (**B**) Quantification showing the relative size of the CFSE-positive area of hMSCs at 1 day and 7 days post-hMSC transplantation. *** *p* < 0.001 vs. CON + hMSCs and ^##^ *p* < 0.005 vs. LPS + hMSCs (1 day) (two-sided paired t-test; *n* = 4 per group). (**C**) Increased expression of MCP-1 and MIP-1α in sites of inflammation is required for effective hMSC migration after LPS exposure. Green indicates CFSE-labeled hMSCs and red indicates MCP-1 and MIP-1α (Scale bar = 50 or 10 μm). hMSCs, human mesenchymal stem cells; CFSE, Carboxyfluorescein Succinimidyl Ester; LPS, lipopolysaccharide; DAPI, 4′,6-diamidino-2-phenylindole; CON, Control.

To effectively deliver hMSCs to injury sites, we injected hMSCs into ICA mice using the intrathecal method, which is a minimally invasive and effective route of administration in the clinical setting. hMSCs were pre-labeled with CFSE dye to investigate their migration. After hMSCs implantation, the overall distribution of hMSCs in the cerebellum was essentially identical in all four ICA mice examined histologically. On day 1, many more clusters of migrated hMSCs were observed in the cerebellar cortex of ICA mice compared to control mice (Figure 2A,B). Most of the hMSCs in ICA mice were distributed in the cerebellar white matter and, to a lesser extent, the external granular layer (EGL) and ependymal surface of the fourth ventricle (Figure 2A). Seven days after the post-transplant, the distribution pattern of hMSCs was similar to that in ICA mice one day after hMSCs transplantation, whereas the hMSC clusters were significantly reduced compared to those on the first day of the post-transplant (Figure 2A,B). In the control group, few hMSCs were observed in the cerebellum and the transplanted hMSCs were preferentially localized in the pia mater, EGL, and ependymal surface of the fourth ventricle in the cerebellum, suggesting less migration of hMSCs in the normal cerebellum.

To investigate whether the enhanced hMSC migration was associated with increased chemoattractants, such as MCP-1 and MIP-1α released by LPS-induced neuroinflammation, we measured the migrated hMSC clusters and chemoattractant expression in the cerebrum and the cerebellum via double immunofluorescence staining. As shown in Figure 2C, the CFSE signals of migrated hMSCs were strongly detected within and near the areas of MCP-1 and MIP-1α positivity in the cerebellum, while the CFSE-labeled hMSCs were not observed in the hippocampus and thalamus of the cerebrum, which were areas of MCP-1 and MIP-1α negativity. These data suggest that the migration of transplanted hMSCs is enhanced by chemoattractants, such as MCP-1 and MIP-1α, secreted in the inflamed cerebellum.

### 3.3. Effects of hMSCs Treatment on the Impaired Behaviors of the LPS-Induced ICA Mice

**Figure 3:**
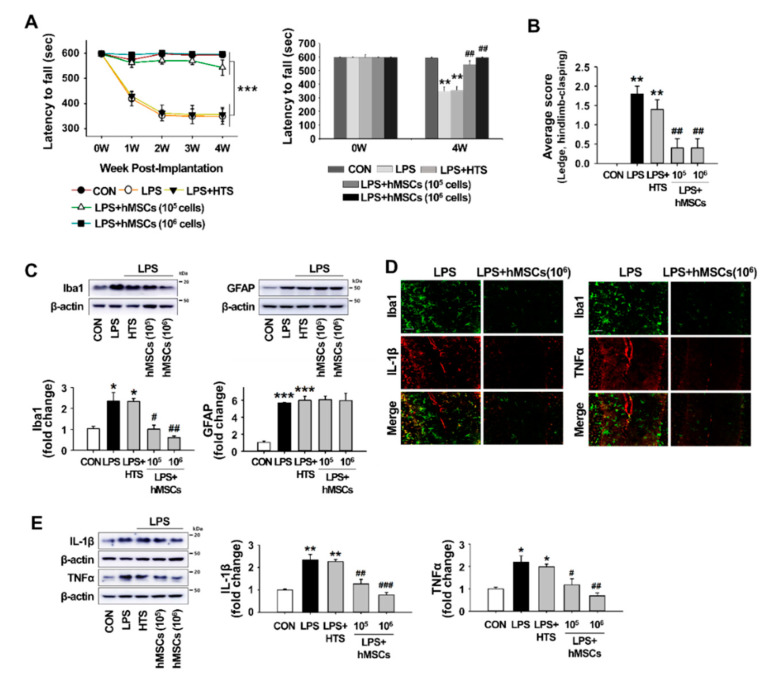

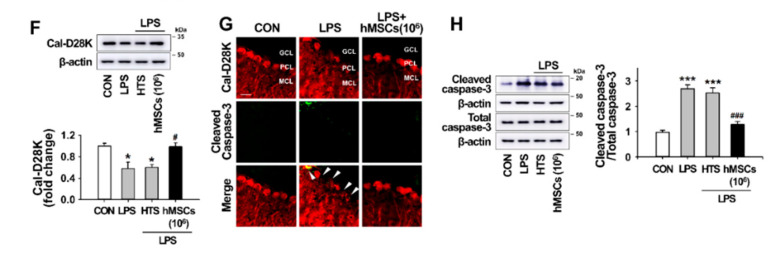
Beneficial effects of hMSCs on behavioral impairments, inflammation, and apoptotic Purkinje cell death induced by LPS. (**A**) Rota-rod test after 4 weeks of hMSC transplantation in ICA mice. Significant differences (*** *p* < 0.001; *n* = 5 per group) between CON or LPS + hMSCs and LPS alone or LPS + HTS groups were found starting at 11 weeks of age and for all testing weeks. ** *p* < 0.01 vs. CON (Mann-Whitney U statistic; *n* = 5 per group) and ^##^ *p* < 0.01 vs. LPS (two-sided paired t-test; *n* = 5 per group). (**B**) Simple composite phenotype scoring system at 4 weeks of post-hMSC transplantation. ** *p* < 0.01 vs. CON (Mann–Whitney U statistic; *n* = 5 per group) and ^##^ *p* < 0.01 vs. LPS (two-sided paired t-test; *n* = 5 per group). (**C**) Western blot analysis of the expression levels of Iba1 (microglia marker) and GFAP (astrocytes marker) in the LPS-exposed cerebellum 7 day after hMSC transplantation. * *p* < 0.05, *** *p* < 0.001 vs. CON and ^#^ *p* < 0.05, ^##^ *p* < 0.01 vs. LPS (*n* = 3 per group). (**D**) The expression pattern of Iba1 (green) and proinflammatory cytokines (IL-1β and TNFα; red) 7 days after hMSC treatment (Scale bar = 50 μm). (**E**) The expression levels of proinflammatory cytokines, such as IL-1β and TNF-α, in the cerebellum at 7 days post-hMSC treatment. * *p* < 0.05, ** *p* < 0.01 vs. CON and ^#^ *p* < 0.05, ^##^ *p* < 0.01, ^###^ *p* < 0.001 vs. LPS (*n* = 3 per group). (**F**) The expression levels of Cal-D28K (Purkinje cell marker) in the cerebellum 7 days after hMSC treatment. * *p* < 0.05 vs. CON and ^#^ *p* < 0.05 vs. LPS (*n* = 4 per group). (**G**) Detection of apoptotic Purkinje cells (arrowheads) in the cerebellum of control, LPS, and hMSC-treated mice 7 days after hMSC treatment, using immunofluorescence analysis of cleaved caspase-3. “MCL” indicating the molecular cell layer, “PCL” indicating the Purkinje cell layer, and “GCL” indicating the granule cell layer (Scale bar = 100 μm). (**H**) Western blot analyses of the expression levels of activated caspase-3 in the cerebellum after 7 days of hMSC treatment. *** *p* < 0.001 vs. CON and ^###^ *p* < 0.001 vs. LPS (*n* = 4 per group). The images of blots were displayed in cropped format. Significance of statistical analysis was analyzed by one-way ANOVA with Tukey’s *post-hoc* analysis (**A**,**C**,**E**,**F**,**H**). hMSCs, human mesenchymal stem cells; CFSE, Carboxyfluorescein Succinimidyl Ester; LPS, lipopolysaccharide; ICA, inflammatory cerebellar ataxias; HTS, HypoThermosol; Iba-1, ionized calcium-binding adapter molecule 1; GFAP, Glial fibrillary acidic protein; CON, Control.

To assess the beneficial effects of intrathecally transplanted hMSCs against CA induced by LPS treatment, we evaluated motor coordination and balance using the rotarod test weekly for 4 weeks after hMSC transplantation, starting at 10 weeks of age (i.e., 1 day before hMSC transplantation). Treatment with HypoThermosol (HTS), an optimized preservation medium used to store stem cells at low temperatures, was performed on the control mice. As shown in Figure 3A, all groups of mice had similar levels of motor coordination before LPS injection. After LPS injection, however, ICA mice exhibited progressive deficits compared to the control mice, and the significant differences between ICA and control mice stared one week after LPS injection, as well as for all testing weeks. The scores in HTS-treated mice were similar to ICA mice, suggesting that HTS does not affect motor performance (Figure 3A). However, the transplantation of both low-dose (1 × 10^5^ cells/mouse) and high-dose (1 × 10^6^ cells/mouse) hMSCs significantly mitigates the mild motor performance decline of ICA mice, although the motor performance associated with low-dose hMSCs tended to be slightly poorer than that associated with high-dose hMSCs four weeks after transplantation. In line with the rotarod test, the composite ataxia phenotype scoring tests demonstrated that hMSC treatment significantly ameliorated the motor impairment and abnormal hindlimb clasping induced by LPS injection (Figure 3B).

### 3.4. Effects of hMSC Transplantation on Neuroinflammation and Purkinje Cell Damage in the Cerebellum of ICA Mice

To investigate the potential effects of hMSCs against neuroinflammation in the cerebellum of ICA mice, we examined whether the treatment with hMSCs inhibited LPS-induced glial activation 7 days after hMSC transplantation, which was the time point indicated by the inflammatory responses following LPS treatment [14]. As shown in Figure 3C, LPS-induced microglial activation was inhibited by hMSC treatment in a dose-dependent manner, whereas reactive astrocytes were not affected by hMSC treatment. The effects of hMSCs on the production of proinflammatory molecules in the cerebellum of ICA mice was evaluated via Western blotting and immunostaining 7 days after hMSC transplantation. The results of double immunostaining showed that the expression of increases in proinflammatory cytokines IL-1β and TNFα level in activated microglia were significantly inhibited by hMSC treatment (Figure 3D). Similar to the results of double immunostaining, Western blotting results demonstrated the significant reduction of both IL-1β and TNFα in the cerebellums of ICA mice by hMSC treatment (Figure 3E).

The effect of hMSCs on Purkinje cell damage caused by LPS-induced neuroinflammation was investigated by the measurement of calbindin levels using Western blotting 7 days after hMSCs transplantation. hMSC treatment prevented LPS-induced loss of Purkinje cells in the cerebellum, while HTS treatment had no effects in decreasing the levels of calbindin in the LPS-injected mice (Figure 3F). Moreover, as shown in Figure 3G,H, LPS-induced cleaved caspase-3 in cerebellar Purkinje cells was significantly inhibited by hMSC treatment, indicating the protection of Purkinje cells through anti-apoptotic cell death.

### 3.5. Effects of hMSCs on Microglial Polarization in the Cerebellum of LPS-Induced ICA Mouse

**Figure 4:**
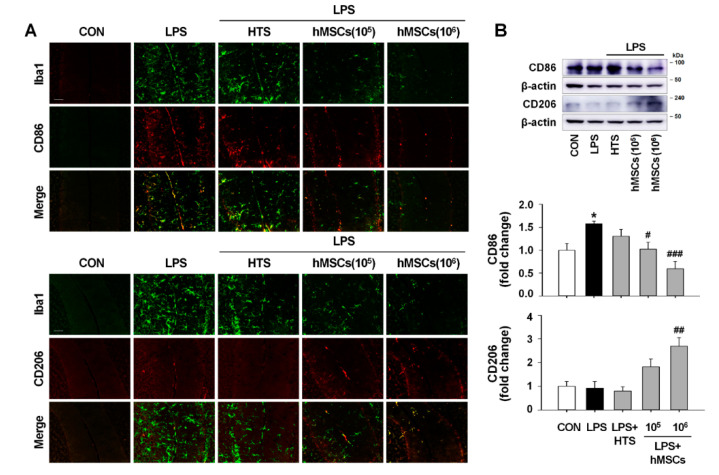

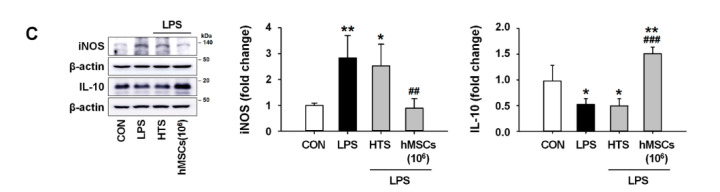
The immunomodulatory effects of hMSCs on regulating microglia polarization in the cerebellum of ICA mice. (**A**) Double immunofluorescence staining for the expression pattern of Iba1 (microglia marker; green) and CD96 (M1-type microglia marker; red), or Iba1 (microglia marker; green) and CD206(M2-type microglia marker; red) in LPS-exposed cerebellum 7 days after hMSC treatment (Scale bar = 50 μm). (**B**) Western blot analyses of the expression levels of CD86 and CD206 in the cerebellum of ICA mice at 7 days post-hMSC transplantation. * *p* < 0.05 vs. CON and ^#^ *p* < 0.05, ^##^ *p* < 0.01, ^###^ *p* < 0.001 vs. LPS (*n* = 3 per group) (**C**) The expression levels of iNOS (M1 marker) and IL-10 (M2 marker) in the LPS-exposed cerebellum 7 days after hMSC transplantation by western blot analyses. * *p* < 0.05, ** *p* < 0.01 vs. CON and ^##^ *p* < 0.01, ^###^ *p* < 0.001 vs. LPS (*n* = 4 per group). The images of blots were displayed in cropped format. Significance of statistical analysis was analyzed by one-one-way ANOVA with Tukey’s post-hoc analysis. hMSCs, human mesenchymal stem cells; CFSE, Carboxyfluorescein Succinimidyl Ester; LPS, lipopolysaccharide; ICA, inflammatory cerebellar ataxias; CON, Control.

We examined the expression levels of M1- and M2-type microglia in order to investigate the effects of hMSCs on the regulation of microglial phenotypes in the cerebellum of ICA mice. Double immunostaining experiments revealed high expression of CD86 (M1 marker) in Iba1-positive microglia following LPS injection, whereas expression of CD206 (M2 marker) was rarely observed in the cerebellum of LPS-injected mice (Figure 4A). However, hMSC treatment induced a significant decrease and increase in CD86 and CD206, respectively, in the LPS-treated cerebellum (Figure 4A). Western blot analyses reveal that LPS exposure in the cerebellum caused significant increase of CD86 expression levels with no alteration of CD206 expression levels compared to intact controls. In addition, hMSC treatment significantly reduced and increased the expression of CD86 and CD206, respectively, compared to levels in LPS-treated ICA mice (Figure 4B). Moreover, in the results of Western blotting for the expression of iNOS and IL-10 as additional M1 and M2 microglial phenotype markers, respectively, in the cerebellum, we observed that LPS-induced expression of iNOS increased (Figure 4C) was inhibited by hMSC treatment (Figure 4C) and that hMSC treatment significantly increased IL-10 expression, which was initially decreased by LPS treatment, compared to levels in the controls (Figure 4C).

### 3.6. TSG-6 Upregulation by hMSC Treatment and its Anti-Inflammatory Effects in the Mouse Cerebellum

**Figure 5:**
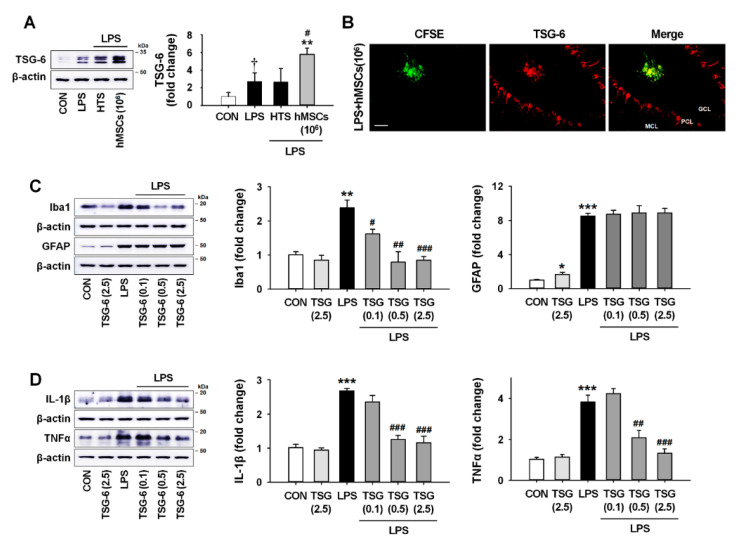
The anti-inflammatory effects of hMSCs-derived TSG-6 in LPS-exposed cerebellum. (**A**) The expression levels of TSG-6 in the LPS-exposed cerebellum 7 days after hMSC transplantation. ** *p* < 0.01 vs. CON and ^#^ *p* < 0.05 vs. LPS (*n* = 3 per group) and ^†^ *p* < 0.05 vs. CON (two-sided paired t-test analysis; *n* = 3 per group). (**B**) The expression pattern of CFSE-labeled hMSCs (green) and TSG-6 (red) in the cerebellum of ICA mice 7 days after hMSC treatment (Scale bar = 50 μm). (**C**) The expression levels of Iba1 and GFAP in the LPS-exposed cerebellum 7 days after rhTSG-6 treatment. * *p* < 0.05, ** *p* < 0.01, *** *p* < 0.001 vs. CON and ^#^ *p* < 0.05, ^##^ *p* < 0.01, ^###^ *p* < 0.001 vs. LPS (two-sided paired t-test analysis, *n* = 4 per group). (**D**) The expression levels of proinflammatory cytokines, such as IL-1β and TNFα, in the cerebellum of ICA mice 7 days after rhTSG-6 treatment. *** *p* < 0.001 vs. CON and ^##^ *p* = 0.01, ^###^ *p* < 0.001 vs. LPS (*n* = 4 per group). The images of blots were displayed in cropped format. Significance of statistical analysis was analyzed by one-one-way ANOVA with Tukey’s post-hoc analysis (**A**,**D**) and two-sided paired t-test (**C**). hMSCs, human mesenchymal stem cells; CFSE, Carboxyfluorescein Succinimidyl Ester; LPS, lipopolysaccharide; ICA, inflammatory cerebellar ataxias; CON, Control.

Although there are few reports of its effects in the cerebellum, TSG-6 is known to be a biomarker of the efficacy of hMSCs for suppressing inflammation in vivo [36,37]. In addition, recent studies showed that TSG-6 secreted by activated glia could act as a modulator for a compensatory mechanism against neurotoxic inflammation [38,39]. To investigate the possibility of an association between hMSC-derived TSG-6 and anti-inflammatory effects of hMSCs in the LPS-induced ICA mice, we analyzed the expression levels of TSG-6 7 days after hMSC treatment. The results obtained by Western blotting and double immunostaining showed that the expression of TSG-6 in astrocytes and Purkinje cells was upregulated after LPS treatment (Figure 5A and Figure S1A,B,C) and that hMSC treatment enhanced the expression of TSG-6 in the ICA mouse cerebellum (Figure 5A,B).

Next, to confirm whether TSG-6 upregulation could induce the anti-inflammatory effects in the LPS-induced ICA mice, we intrathecally injected recombinant human TSG-6 (rhTSG-6) at different concentrations (0.1, 0.5, and 2.5 μg), instead of hMSC, into the cisterna magna, and then measured the expression levels of Iba1, GFAP, IL-1β and TNFα by western blotting at 7 days after treatment. The results showed significant dose-dependent decreases in Iba1 expression in the presence of rhTSG-6 compared to LPS alone (Figure 5C). GFAP expression had no significant difference between the LPS-injected and the rhTSG-6-treated groups. In addition, treatment with 0.5 and 2.5 μg rhTSG-6 significantly inhibited the expression of IL-1β (Figure 5D) and TNFα (Figure 5D), suggesting that TSG-6 upregulation by hMSC treatment contributed to the inhibition of inflammatory responses in the LPS-induced ICA mice.

## 4. Discussion

CAs are movement disorders originating from a cerebellum damaged by one or a combination of various causes [1,3,6]. CAs characterized by uncoordinated muscle movement can be classified into three types: idiopathic, acquired, and hereditary [40]. Recently, accumulated evidence demonstrates that cerebellar inflammation contributes to the progression of CAs. For example, many types of acquired ataxia (e.g., toxin-induced cerebellar ataxia and immune or infection-mediated cerebellar ataxia) are known to be closely associated with cerebellar inflammation [2]. Moreover, the production of inflammatory mediators in over-activated glial cells as part of the neurotoxic inflammatory responses are observed in patients and in animal models of spinocerebellar ataxia [8,9,10]. Additionally, the cortex neurons and Purkinje cells in cerebellum are particularly vulnerable to poisoning [41]. Therefore, neurotoxic inflammation caused by toxic exposure such as drugs, alcohol, and environmental chemicals can cause CAs [11,12,42]. These findings suggest that neuroinflammation in cerebellum is an important risk factor in the progression of CAs and an attractive therapeutic target for the treatment of CAs.

The fundamental mechanism of neurodegenerative diseases, including CAs, is the progressive functional and quantitative loss of neurons. Unfortunately, there are still no effective treatments to delay the progressive neurodegeneration of CA [3,43]. However, stem cell therapies, especially with MSCs, hold great promise for the treatment of neurological diseases [43,44]. Recently, preclinical and clinical studies addressing MSCs to treat neurodegenerative diseases have exponentially increased because of their anti-inflammatory function, regeneration capacity, and low immunogenicity [45]. However, there is no previous study of the effects of MSCs in an animal model of CA associated with neuroinflammation. To assess the therapeutic effects of hMSCs on inflammatory CAs (ICA), we used ICA mice, which were developed in our previous study [14]. A previous study of ICA mice showed that balance and motor control disabilities, indicating cerebellar dysfunction, were accompanied by the upregulation of proinflammatory molecules via glial activation and followed by apoptotic Purkinje cell loss. These results demonstrate that ICA mice exhibit inflammatory responses and symptoms typical of CA patients and are thus a viable experimental animal model of ICA. First, we confirmed that cells used in all experiments exhibited typical hMSC characteristics, including a spindle-shaped fibroblastic morphology, surface marker expression, and multi-lineage differentiation (Figure 1). This study focused on inhibition of the inflammatory response of cerebellar ataxia by hMSC secretion without confirming the therapeutic effect of cell differentiation. Further studies are needed to clarify what types of cells can be differentiated into and what types of cell populations can be altered by administration of multipotent hMSCs in ICA mice. To assess the beneficial effects of hMSCs in inflammatory CA, we then analyzed the anti-inflammatory effects of transplanted hMSCs, delivered intrathecally through the cisterna magna to increase their therapeutic potential in the cerebellum, in LPS-induced ICA mice. After hMSC transplantation, cells specifically migrated to the LPS-treated mouse cerebellum (demonstrated by comparison with non-LPS exposed controls), particularly to the cerebellar cortex and white matter, and a small number of migrated hMSCs were preserved until 7 days after transplant (Figure 2A,B). Furthermore, hMSC clusters were predominantly located near the areas positive for MCP-1 and MIP-1α upregulated by LPS treatment (Figure 2C), as in the results of a previous study [14]. Our results accord with those of previous studies, suggesting that inflammatory chemokines strongly stimulate the migration of MSCs to damaged regions in neurodegenerative diseases [46,47,48]. Thus, our results suggest that MCP-1 and MIP-1α, as chemoattractant molecules associated with inflammatory responses in the LPS-treated cerebellum, may play important roles in the migration of hMSCs to the inflamed cerebellum of ICA mice.

Many anti-inflammatory actions of hMSCs have been previously demonstrated in neurodegenerative disorders, such as Alzheimer’s [49], Huntington’s [50], and Parkinson’s disease [51]. Indicating the therapeutic potential of hMSCs in ICA, LPS-treated ICA mice that received intrathecal transplantation of hMSCs had significantly improved rotarod phenotypes, as well as enhanced motor coordination and hindlimb clasping compared to LPS-treated mice who did not receive hMSCs (Figure 3A,B). Further, Western blotting to assess the pathological alterations associated with glial activation showed that hMSC treatment significantly suppresses LPS-induced microglial activation in the cerebellum (Figure 3C). However, the levels of GFAP, indicating astroglial activation, were not affected by hMSC treatment (Figure 3C). Consistent with the levels of Iba1-positive microglia (Figure 3C), the increased levels of IL-1β and TNFα in activated microglia following LPS exposure were significantly inhibited by hMSC treatment (Figure 3D,E).

In neurodegenerative disorders, microglia activation is an integral feature of neuroinflammation, leading to progressive brain damage. However, changes in the phenotype and profile of microglia during the process of neuroinflammation under specific disease are still a controversial issue [52]. Depending on the types of pathologic events and stimuli, activated microglia are categorized into two phenotypes with different function: M1 phenotype related to proinflammatory responses and M2 phenotype corresponding with anti-inflammatory responses. Recent studies indicate the neurotoxic properties of microglia, whereas others show the beneficial effects of microglia on the neurons in certain circumstances through the removal of toxic proteins and the secretion of neurotrophic factor [53]. Although, M1-type microglia as a resistance barrier eliminates foreign invasion of pathogens or removes toxic protein and cell debris, it also accelerates neuron damages and neurotoxic inflammation in CNS [54]. Recently, hMSCs have been reported to promote microglial polarization toward the M2 phenotype [55,56]. The cell surface markers and cytokine expression levels of M1- and M2-type microglia 7 days after hMSC treatment in the cerebellum of ICA mice indicate that most activated microglia were polarized toward an M1 phenotype after LPS exposure, which was demonstrated by the increased CD86 and iNOS levels (Figure 4). However, hMSC treatment induced microglial polarization toward an M2 phenotype, demonstrated by the decreased levels of CD86 and iNOS and by the increased levels of CD206 and IL-10 (M2 markers) (Figure 4). Together with the evidence of inhibition of the proinflammatory molecules IL-1β and TNFα (Figure 3D,E), these results suggest that hMSCs induce beneficial alteration of activated microglia, resulting in the induction of anti-inflammatory effects in the LPS-treated cerebellum.

As previously reported, TSG-6 derived from hMSCs can inhibit inflammatory responses in various organs, which may be beneficial for the treatment of diseases [57,58,59]. Moreover, hMSCs inhibit LPS-induced inflammatory responses through the release of TSG-6 in vitro [55,56], suggesting that TSG-6 upregulation might be useful as a biomarker to predict the efficacy of hMSCs in suppressing inflammation. The results of our experiments on the effects of hMSC-derived TSG-6 against neuroinflammation in the ICA mouse cerebellum reveal that hMSC treatment upregulates the protein levels of TSG-6 (Figure 5A,B) and that treatment with rhTSG-6 significantly inhibits the expression of IL-1β and TNFα by activated microglia following LPS exposure (Figure 5C,D). These findings suggest that TSG-6 upregulation might be a key mechanism of the hMSC-induced anti-inflammatory effects in the LPS-treated cerebellum.

Along with these anti-inflammatory effects, hMSCs have neuroprotective effects in vitro, mitigating neuronal apoptosis, parthanatos, and necroptosis in hypoxic-ischemic injury [60]. Similar to these previous findings, our results show that hMSC administration in the LPS-exposed mouse cerebellum inhibits Purkinje cell death, which is a pathological feature of CA (Figure 3F–H). Further studies are needed to clarify whether the loss of Purkinje cells was caused solely by neuroinflammation following LPS treatment or also involved a non-apoptotic cell death mechanism besides apoptosis. Nevertheless, our observations demonstrate that the improvement of voluntary movement coordination in LPS-induced ICA mice may be due to the inhibition of Purkinje cell death through the anti-inflammatory effects exerted by hMSC-derived TSG-6.

## 5. Conclusions

**Figure 6:**
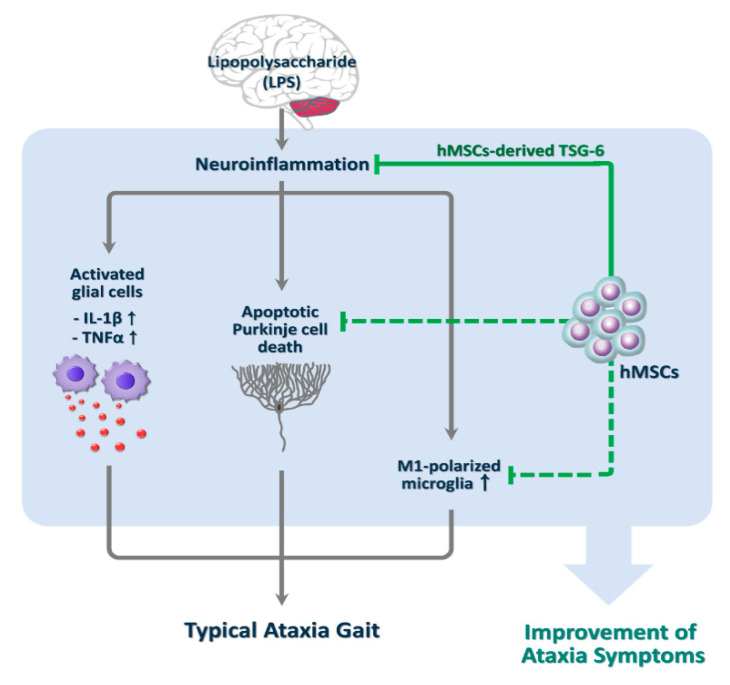
Schematic for the beneficial effects of hMSCs in the LPS-induced ICA mice. Intercerebellar LPS administration in experimental animals may be useful to study the phenotypes of ICA, and hMSCs may have therapeutic effects though the inhibition of neurotoxic inflammation, M1-type microglial polarization, and apoptotic Purkinje cell death against ICA, resulting in the improvement of ataxia symptoms. hMSCs, human mesenchymal stem cells; ICA, inflammatory cerebellar ataxias.

In summary, we investigated whether hMSCs have potential beneficial effects against neuroinflammation-induced CA, using ICA mice, which previously reported that LPS-induced ICA mice induced apoptotic death of Purkinje cells caused by upregulation of proinflammatory molecules via glial activation, resulting in impairments of balance and motor control (Figure 6) [14]. Our results show that hMSC treatment significantly ameliorated ataxia symptoms in the LPS-induced ICA mice, and the beneficial effects were related to the inhibition of glial activation, resulting in decreased levels of proinflammatory molecules and the prevention of apoptotic Purkinje cell death (Figure 6). Taken together, although it is needed to further examine whether hMSCs directly affected to the inhibition of apoptotic Purkinje cell death and M1-type microglial polarization in the LPS-treated cerebellum, these findings suggest that hMSC therapies could provide an effective therapeutic approach to ICA through the inhibition of neurotoxic inflammation mediated by M2-type microglial polarization and TSG-6 upregulation and through the prevention of apoptotic Purkinje cell death.

## Figures and Tables

**Table 1:** List of antibody used for immunofluorescence (IF) staining and western blot (WB).

Antibody Name	Source	Identifier	Dilution
Rabbit anti-ionized calcium-binding adapter molecule 1	Wako Pure Chemical Industries, Osaka, Japan	019-19741	IF 1:2000
			WB 1:1500
Rabbit anti-glial fibrillary acidic protein	Millipore, Billerica, MA, USA	AB5804	IF 1:2000
			WB 1:2000
Rabbit anti-interleukin 1 beta	Abcam, Cambridge, UK	Ab9722	IF 1:500
			WB 1:1000
Mouse anti-tumor necrosis factor alpha	Abcam, Cambridge, UK	Ab6671	IF 1:500
			WB 1:1000
Rat anti-monocyte chemoattractant protein 1	Abcam, Cambridge, UK	Ab8101	IF 1:100
			WB 1:500
Rat anti-macrophage inflammatory protein 1 alpha	R&D Systems, Minneapolis, MN, USA	MAB4501	IF 1:100,
			WB 1:1000
Mouse anti-calbindin-D-28K	Sigma, St. Louis, MO, USA	C9848	IF 1:500
			WB 1:2000
Rabbit anti-cleaved caspase-3	Cell Signaling, Beverly, MA, USA	CST 9664	IF 1:400
			WB 1:1000
Rabbit anti-caspase-3	Cell Signaling, Beverly, MA, USA	CST 9662	WB 1:1000
Rabbit anti-cluster of differentiation 86	Invitrogen, Carlsbad, CA, USA	14-0863-81	IF 1:200,
			WB 1:1000
Goat anti-cluster of differentiation 206	R&D Systems, Minneapolis, MN, USA	AF2535	IF 1:200
			WB 1:1000
Rabbit anti-inducible nitric oxide synthase	Abcam, Cambridge, UK	Ab3523	WB 1:1000
Rabbit anti-interleukin 10	Abcam, Cambridge, MA, USA	Ab9969	WB 1:1000
Rabbit anti-TNFα stimulated gene-6	GeneTex, Irvine, CA, USA	GTX55175	WB 1:1000
Mouse anti-β-actin	Santa cruz biotechnology, Santa Cruz, CA, USA	sc-47778	WB 1:2000
Texas Red-conjugated anti-mouse IgG antibody	Vector Laboratories, Burlingame, CA, USA	TI-2000	IF 1:400
Texas Red-conjugated anti-Goat IgG antibody	Vector Laboratories, Burlingame, CA, USA	TI-5000	IF 1:400
FITC-labeled IgG antibody	Jackson ImmunoResearch, West Grove, PA, USA	FI-2000	IF 1:400
horseradish peroxidase (HRP)-conjugated antibody	Thermo Fisher Scientific, Piscataway, NJ, USA	61-6520	WB 1:4000

## Supplementary Materials

The following are available online at https://www.mdpi.com/2077-0383/9/11/3654/s1, Figure S1: TSG-6 upregulation in the LPS-exposed cerebellum.
