# Supplementary material for: Therapeutic Effects of Human Mesenchymal Stem Cells in a Mouse Model of Cerebellar Ataxia with Neuroinflammation
Source: J Clin Med. 2020 Nov 13;9(11):3654. doi: 10.3390/jcm9113654 (PMC7698164; doi:10.3390/jcm9113654)
Supplement: Supplementary file 1 [file jcm-09-03654-s001.pdf]

Supplementary Materials

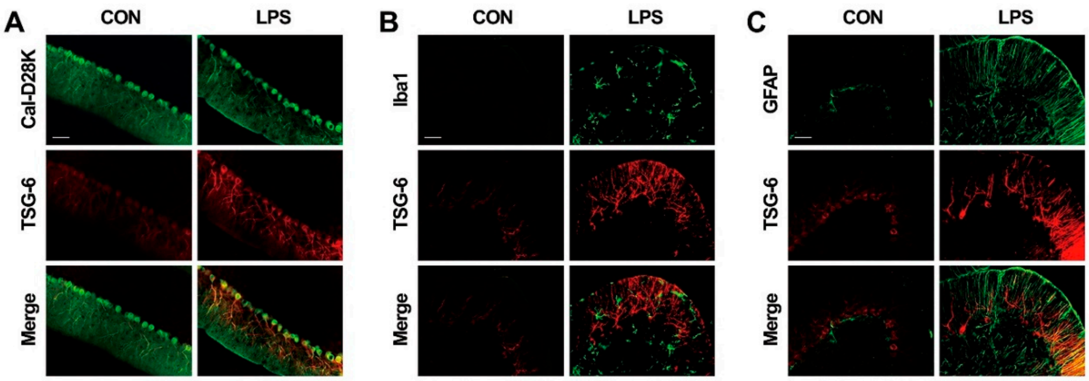

Figure 1. TSG-6 upregulation in the LPS-exposed cerebellum. (**A**-**C**) The expression patterns of TSG-6 (red) and various cerebellar cells (green), including Purkinje cells (**A**), microglia (**B**), and astrocytes (**C**), 7 days after LPS treatment in the cerebellum of a mouse's brain. TSG-6 expression increased in the cerebellum after LPS injection and mainly colocalized with astrocytes and Purkinje cells. Scale bar = 50 μm.
